# AKAP5 and AKAP12 Form Homo-oligomers

**DOI:** 10.1186/1750-2187-6-3

**Published:** 2011-05-09

**Authors:** Shujuan Gao, Hsien-yu Wang, Craig C Malbon

**Affiliations:** 1 Departments of Pharmacology, Heath Sciences Center, School of Medicine, State Univerdity of New York at Stony Brook, NY 11794-8651 USA; 2 Physiology & Biophysics, Health Sciences Center, School of Medicine, State Univerdity of New York at Stony Brook, NY 11794-8661 USA

## Abstract

**Background:**

A-kinase-anchoring proteins, AKAPs, constitute a family of scaffolds that play an essential role in catalyzing the spatial-temporal, dynamic interactions of protein kinase A, protein kinase C, tyrosine kinases, G-protein-coupled receptors and ion channels. We studied AKAP5 (AKAP79; MW ~47 kDa) and AKAP12 (gravin, SSECKS; MW ~191 kDa) to probe if these AKAP scaffolds oligomerize.

**Results:**

In gel analysis and sodium-dodecyl sulfate denaturation, AKAP12 behaved with a MW of a homo-dimer. Only in the presence of the chaotropic agent 8 M urea did gel analysis reveal a monomeric form of AKAP12. By separation by steric-exclusion chromatography, AKAP12 migrates with MW of ~840 kDa, suggestive of higher-order complexes such as a tetramer. Interestingly, the N-(1-840) and C-(840-1782) terminal regions of AKAP12 themselves retained the ability to form dimers, suggesting that the structural basis for the dimerization is not restricted to a single "domain" found within the molecule. In either sodium dodecyl sulfate or urea, AKAP5 displayed a relative mobility of a monomer, but by co-immunoprecipitation in native state was shown to oligomerize. When subjected to steric-exclusion chromatography, AKAP5 forms higher-order complexes with MW ~220 kDa, suggestive of tetrameric assemblies.

**Conclusion:**

Both AKAP5 and AKAP12 display the capacity to form supermolecular homo-oligomeric structures that likely influence the localization and function of these molecular scaffolds.

## Background

Discovery of a docking site for the regulatory subunits (*i.e*., RI/RII) of cyclic AMP-dependent protein kinase A (PKA, A-kinase) was seminal in our understanding of the roles of A-kinase-anchoring protein (AKAP) scaffolds in cellular signaling [1-4]. AKAPs not only dock PKA, but act as molecular "tool boxes" that are multivalent and capable of docking other protein kinases (protein kinase C and the tyrosine kinase Src), phosphoprotein phosphatases, such as protein phosphatase 2B (PP2B) [5], cyclic AMP phosphodiesterases (PDE) [6-9], adaptor molecules [8-11], ion channels [12-14], and members of the superfamily of G protein-coupled receptors (GPCR) [15-17]. Two AKAPs, AKAP5 and AKAP12, that associate with the prototypic GPCR, the β_2_-adrenergic receptor (β_2_-AR), have been the focus of intense research [16,18-21]. Herein, we examine these two members of the class of GPCR-associated AKAPs and explore the extent to which these proteins, whose structure is predicted to be natively unordered [5], are capable of forming oligomers. The current work is the first to report that both AKAP5 and AKAP12 are not only capable of forming homodimers, but also of forming higher-order supermolecular homo-oligomeric complexes. Thus AKAP oligomerization adds a new dimension on how members of this class of scaffold molecules operate in cell signaling.

## Results

### Oligomerization of AKAP12

**Figure 1:**
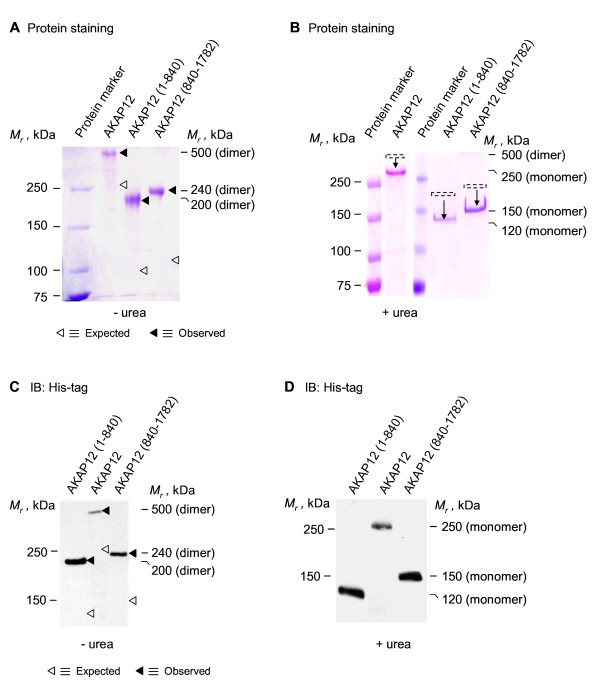
**Oligomerization of AKAP12 (and either C-terminal or N-terminal fragments) expressed and purified from *E. coli***. His-tagged AKAP12 or His-tagged AKAP12 fragments were expressed in *E. coli*. The AKAP12 (or fragments) were purified and then resolved on SDS-PAGE in the absence (A) or presence of 8 M urea (B). The gels were fixed and stained for protein with Coomassie Brilliant Blue (A, B) or the resolved proteins transferred to PVDF membrane and subjected to immunoblotting (C, D). The "◀" indicates the observed *M_r _*on the gels and the "Δ" indicates the predicted *M_r_*, calculated from the primary sequence.

We first sought to interrogate if oligomerization of AKAP12 could be detected using purified human AKAP12. Consequently, His-tagged human AKAP12 was expressed in *E. coli*, purified by gel filtration chromatography, and analyzed by SDS-PAGE. Analysis of the expressed, purified AKAP12 in the presence of the denaturant SDS revealed a *M_r _*of ~500 kDa, slightly more than twice the mass of that calculated (hAKAP12, MW = 191.5 kDa) based upon primary sequence alone (see protein staining, Figure 1A). The nature of the 500 kDa-*M_r _*species was made clear by performing the same analysis, but with prior sample treatment and SDS-PAGE separation performed in the presence of the chaotropic agent 8 M urea (Figure 1B). In the presence of 8 M urea, the apparent *M_r _*of purified AKAP12 declined from ~500 kDa to ~250 kDa, emblematic of a monomeric form of this large AKAP. The results obtained of fixed gels, stained for protein (Figure 1A, B) were further tested using immunoblotting to detect the protein (Figure 1C, D). Immunoblots of the resolved, transferred protein that were stained with antibodies to the His-tag confirmed the results of the protein staining (Figures 1C, D). AKAP12 forms oligomers that are SDS-resistant. Homodimeric AKAP12 can only be disassembled by treatment and separation in 8 M urea, a strong chaotropic agent.

**Figure 2:**
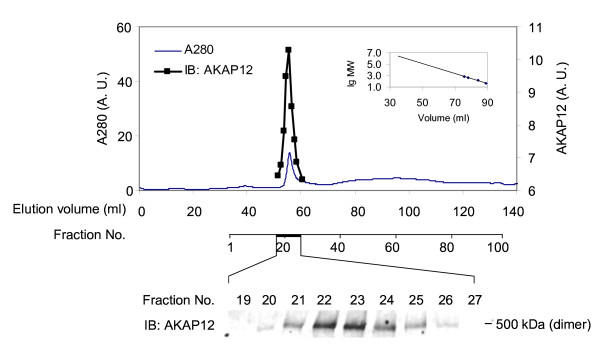
**Steric-exclusion chromatography of purified AKAP12**. His-tagged AKAP12 was purified and then resolved by steric-exclusion chromatography performed on an AKTA FPLC fitted with HiPrep Sephacryl-S400 (16/60) column. The presence of supermolecular oligomers of AKAP12 was established by SDS-PAGE and immunoblotting of samples from the chromatography. The resolved, transferred proteins were stained with anti-AKAP12 antibodies. Marker protein mobilities were employed to establish the *M_r _*of AKAP12 by elution position (inset). The results displayed are representative of three separate experiments performed on as many separate cell cultures.

Since we established the existence of AKAP12 oligomers using SDS-PAGE, we probed the upper limit size of AKAP12 oligomers. AKAP12 purified from *E. coli *was subjected to steric-exclusion chromatography on a high-resolution, wide-bore matrix operated by an AKTA FPLC system (Figure 2). A spark symmetrical peak of protein (A280) and of AKAP12 immunoreactivity (see blot inset) was resolved. Using a set of marker proteins of known *M_r_*, we were able to size the AKAP12 supermolecular complexes with precision. The *M_r _*of the AKAP12 was 830-850 kDa. AKAP12 appears to form oligomers greater than dimers, minimally trimeric or even tetrameric. This higher order assembly of AKAPs into supermolecular complexes is a novel observation.

**Figure 3:**
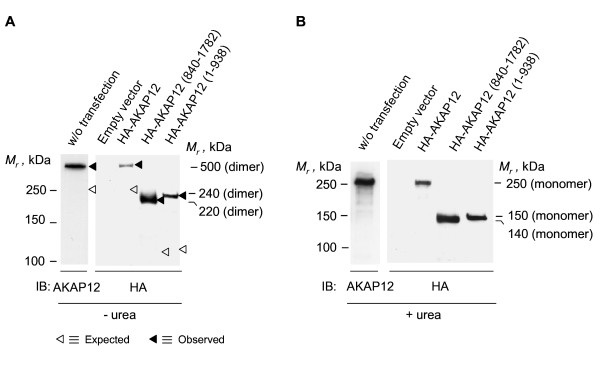
**Oligomerization of AKAP12 (and either C-terminal or N-terminal fragments) expressed in mammalian HEK293 cells**. Human embryonic kidney (HEK) 293 cells were transfected with an empty expression vector or one harboring either full-length AKAP12, AKAP12 N-terminal (1-938) or C-terminal (840-1782) regions. Whole-cell lysates of cells expressing either AKAP12 or one of the fragments were subjected to SDS-PAGE in the absence (A) or presence of 8 M urea (B). The resolved proteins were transferred to PVDF membrane and subjected to immunoblotting, stained for AKAP12 or the HA-tag. The results displayed are representative of at least three separate experiments performed on as many separate cell cultures.

We probed the same queries for AKAP12 expressed in mammalian cells. Human embryonic kidney 293 (HEK293) cells transfected with an expression vector harboring the human AKAP12 were collected, lysed, and the mobility of AKAP12 established by SDS-PAGE (Figure 3A). Homodimers of HA-tagged AKAP12 exogenously expressed in HEK293 cells were found to be resistant to SDS denaturation. By SDS-PAGE analysis, a ~500 kDa-*M_r _*immunoreactive band was obvious, whether the immunoblots were stained with anti-AKAP12 or anti-HA antibodies (Figure 3A). When the samples were treated with and subjected to SDS-PAGE in the presence of 8 M urea, the *M_r _*of the immunoreactive band of AKAP12 declined from ~500 kDa to ~250 kDa (Figure 3B). This ~250 kDa mobility is consistent with the MW calculated for the monomeric form of the scaffold protein from primary sequence.

We investigated if N-terminal (1-840) and C-terminal (840-1782) fragments of AKAP12 would retain the ability to form dimers, as observed with the full-length AKAP12 (Figure 1A, B). Expression vectors harboring either the His-tagged N-terminal or the C-terminal region of AKAP12 were constructed, the protein was expressed in *E. coli*, and each of the two large fragments was purified by affinity chromatography. Protein staining of the N- and C-terminal fragments resolved by gel analysis in the presence of SDS reveals, remarkably, the homodimerization of both the N-terminal as well as the C-terminal fragments of AKAP12 (Figure 1A). In the presence of 8 M urea, however, the N-terminal and C-terminal fragments of AKAP12 now can be resolved as monomeric forms (Figure 1B). Immunoblotting of the His-tagged fragments resolved in SDS (Figure 1C, D) confirms the identity of the species made visible by protein staining (Figure 1A, B), *i.e*., both AKAP12 fragments behave as dimers in SDS-PAGE. In the presence of 8 M urea, the N- and C-terminal fragments made visible by protein staining (Figure 1B) likewise resolve as the monomeric forms, as made visible by immunoblotting (Figure 1C, D).

The electrophoretic behavior of full-length human AKAP12, as well as the N- and the C-fragments was probed when these fragments were expressed in mammalian (HEK293) cells, rather than in *E. coli*. The expressed HA-tagged versions of the full-length AKAP12 (1-1782), the N-terminal fragment (1-938), and a C-terminal (840-1782) fragment of AKAP12 were subjected to SDS-PAGE, immunoblotting of the resolved proteins, and made visible by staining with anti-HA antibody (Figure 3A, B). In the presence of SDS, HA-tagged AKAP12 displays a *M_r _*of a dimer (Figure 3A). Immunostaining of the HEK cell extracts with anti-AKAP12 antibody likewise displays the same homodimeric character. When treated and resolved on gels in the presence of 8 M urea, either exogenously (probed with anti-HA immunostaining) or endogenously (probed with anti-AKAP12 antibody) expressed AKAP12 migrated with a *M_r _*of a monomer (Figure 3B). Both the N-terminal as well as the C-terminal fragments of AKAP12 expressed in mammalian cells displayed dimers in the presence of SDS (Figures 1A, 3A) and monomers in the presence of 8 M urea (Figures 1B, 3B). Thus we establish that the dimeric character of AKAP12 and these two large AKAP fragments reflects neither the presence/absence of tags nor whether the proteins are exogenously expressed in *E. coli *or endogenously in mammalian cells, i.e., native AKAP12 oligomerizes.

**Figure 4:**
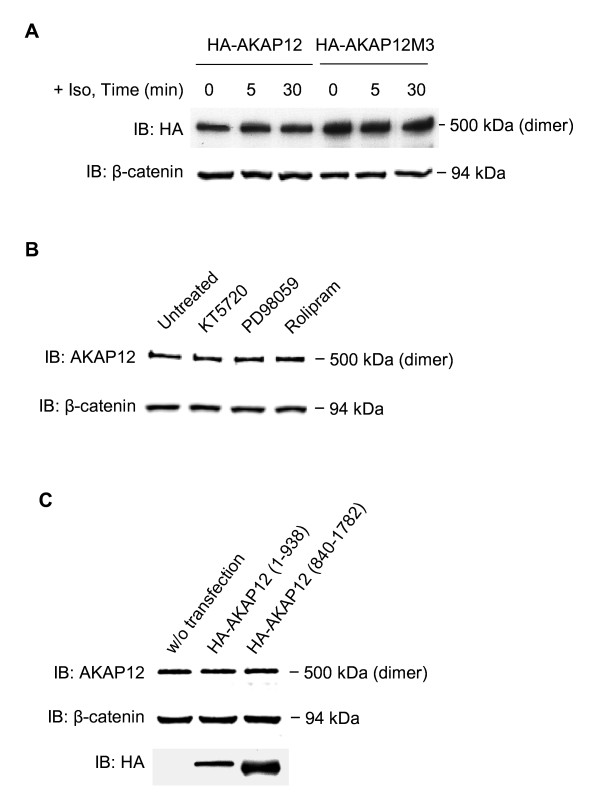
**Oligomerization of AKAP12 in mammalian cells: elevation of intracellular cyclic AMP as well as inhibition of protein kinase A, MEK1/2, cyclic AMP phosphodiesterase 4, or expression of either N- or C-terminal fragments do not block AKAP oligomerization**. (A) HEK293 cells were transfected with an expression vector harboring either HA-tagged full-length AKAP12 (HA-AKAP12) or an HA-tagged AKAP12 in which alanine substitution of protein kinase A phosphorylation sites (S627A, S696-698A, S772A) has been performed (AKAP12M3). Cells were treated with the beta-adrenergic agonist isoproterenol (10 μM) for 0, 5, or 30 min prior to harvesting the cells for lysis. Whole-cell lysates of transfected cells expressing either AKAP12 or AKAP12M3 were subjected to SDS-PAGE in the absence of 8 M urea. (B) HEK293 cells were either untreated or incubated with a chemical inhibitor for protein kinase A (KT5720, 1 μM), MEK1/2 (PD98059, 20 μM), or cyclic AMP phosphodiesterase 4 (Rolipram, 10 μM) for 45 min prior to cell lysis and subsequent analysis by SDS-PAGE. (C) HEK293 cells were either untransfected or transfected with an expression vector harboring the AKAP12 N-terminal (1-938) fragment or the C-terminal (840-1782) fragment. Whole-cell lysates of cells expressing either AKAP12 or one of the fragments subjected to SDS-PAGE in the absence of 8 M urea. The resolved proteins were transferred to PVDF membrane, subjected to immunoblotting, and made visible by staining with anti-HA or anti-AKAP12 antibodies. Beta-catenin was employed as a loading control for each lane, also identified by immunoblotting, stained with anti-beta-catenin antibodies. The results displayed are representative of at least three separate experiments performed on as many separate cell cultures.

Since AKAP12 mediates recycling and resensitization of beta-adrenergic receptors [4,5,22-26], which mediate elevation of intracellular cyclic AMP, we sought to interrogate the effects of modulating cyclic AMP signaling pathway on the oligomerization of AKAP12 (Figure 4). Stimulation of cyclic AMP accumulation in HEK293 cells by stimulation of the cells with beta-adrenergic agonist, isoproterenol, for 5 or 30 min had little effect on the mobility of AKAP12 on SDS-PAGE, ~500 kDa-*M_r _*(Figure 4A). To test this possibility further, we examined the effect of mutating the three sites of protein kinase A-catalyzed phosphorylation of AKAP12 on the ability of the AKAP12 to form oligomers [22]. AKAP12 mutated by alanine substitution of protein kinase A phosphorylation sites (Ser627Ala, Ser696-698Ala, Ser772Ala, labeled "AKAP12M3") was expressed in HEK293 cells, the cells treated without or with isoproterenol, and the *M_r _*of AKAP12 established by SDS-PAGE (Figure 4A). AKAP12M3 displayed the same *M_r _*(~500 kDa) as native AKAP12, i.e., no change in dimer formation in response to stimulation of the cells with isoproterenol. Cells were then challenged with inhibitors to protein kinase A (KT5720), but also to cyclic AMP phosphodiesterase 4 (PDE4, Rolipram), and to MEK1/2 (PD98059). The first two inhibitors test cyclic AMP signaling by blocking protein kinase A and PDE4, respectively, The third inhibits MEK1/2, the enzyme through which the mitogen-activated kinase cascade (i.e., Erk1/2 activation) can be stimulated by beta-adrenergic activation, an AKAP-sensitive response (Figure 4B). Treating the cells with any one of these specific enzyme inhibitors did not noticeably alter AKAP12 oligomerization, i.e., the *M_r _*(~500 kDa) of AKAP12 on SDS-PAGE was unaffected. Finally, we tested if the overexpression of either the N-terminal (1-938) or the C-terminal (840-1782) fragments in HEK293 cells would affect the formation of the SDS-resistant dimers of full-length endogenous AKAP12 (Figure 4C). The presence of neither fragment influenced the formation of the SDS-resistant dimers (*M_r _*~500 kDa) of AKAP12. Both the N-terminal and C-terminal fragments of AKAP12 expressed well in the HEK293 cells, although the expression of the later was more robust (Figure 4C).

### Oligomerization of AKAP5

**Figure 5:**
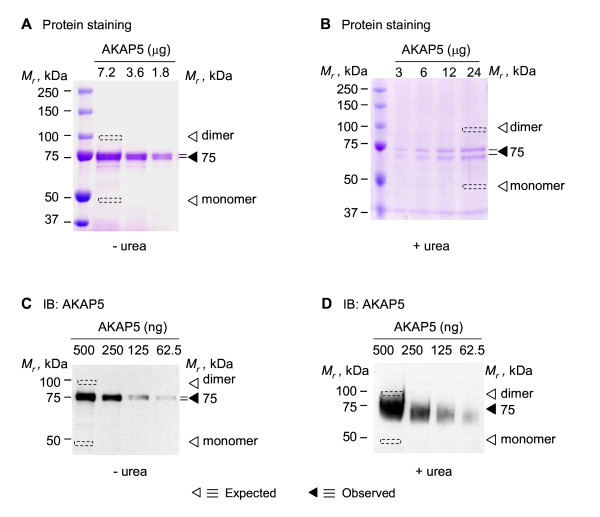
**Oligomerization of AKAP5 expressed and purified from *E. coli***. His-tagged AKAP5 was expressed in *E. coli*. Affinity purified AKAP5 was resolved on either SDS-PAGE (A, C) or SDS-PAGE performed in the presence of 8 M urea (B, D). The gels were fixed and stained for protein with Coomassie Brilliant Blue (A, B) or the resolved proteins transferred to PVDF membrane and subjected to immunoblotting (C, D). Immunoblotting was performed using anti-AKAP5 antibody (C, D). The "◀" indicates the observed *M_r _*on the gels and the "Δ" indicates the predicted *M_r_*, calculated from the MW (47 kDa).

We sought to examine if the oligomeric behavior observed for AKAP12 would be observed in a second, much smaller, AKAP that docks to GPCRs, the AKAP5 (a.k.a., AKAP79; MW = 47.1 kDa versus 191.5 kDa for AKAP12, based upon primary sequence). His-tagged human AKAP5 was expressed in *E. coli*, purified by affinity chromatography, and subjected to to SDS-PAGE in the absence or presence of 8 M urea (Figure 5). In the presence of either denaturant, AKAP5 behaves as a monomer, although often as a doublet with *M_r _*~75 kDa (Figure 5A, B). The microheterogenity of AKAP5 reflects, perhaps, differences in some post-translational modifications, particularly protein phosphorylation. The results of the protein staining were tested by immunoblotting of the AKAP with anti-AKAP5 antibody (Figure 5C, D). The immunoblotting and protein staining data for AKAP5 mobility on gels are in good agreement. Formation of SDS-resistant AKAP5 oligomers, as we had observed for AKAP12, was not observed.

**Figure 6:**
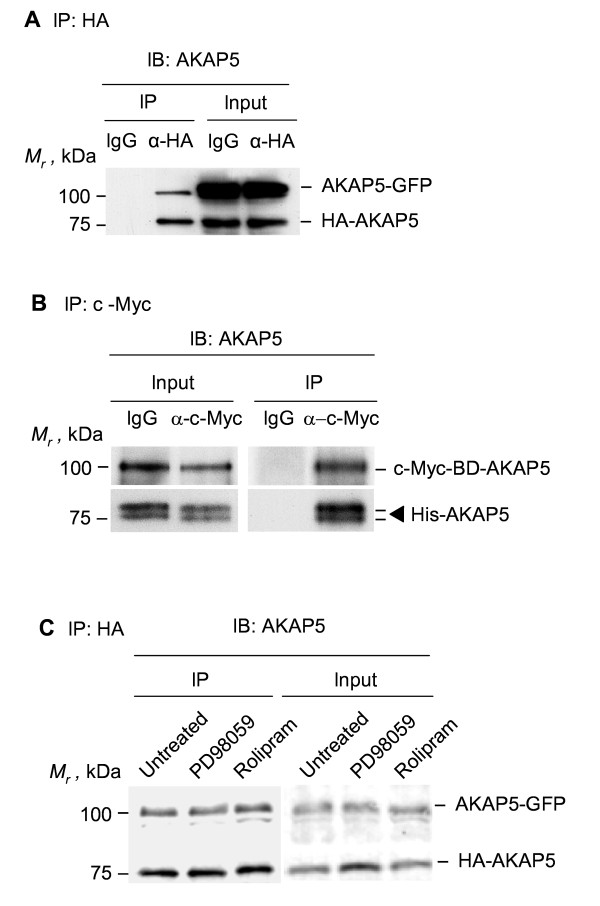
**Oligomerization of AKAP5 *in vivo *and *in vitro***. (A) HEK293 cells stably expressing GFP-tagged AKAP5 were transiently transfected with HA-tagged AKAP5. Cell lysates were subjected to pull-downs mediated by IgG (control) or anti-HA antibodies (IP-HA) and the pulldowns subjected to SDS-PAGE, immunoblotting, and stained with anti-AKAP5 antibodies. Endogenous AKAP5 (expressed at <10% of that of exogenously expressed, tagged-AKAP) display a *M_r _*identical with the HA-tagged version under these conditions. (B) c-Myc-tagged AKAP5 fused to amino acids 1-147 of the GAL4 DNA-binding domain (BD) was expressed in yeast. The c-Myc tagged AKAP5 was subjected to pull-down with anti-c-Myc antibody or control IgG. The immune complexes then were incubated with purified His-tagged AKAP5. The association of His-tagged AKAP5 with the c-Myc-tagged AKAP5 was detected by SDS-PAGE, immunoblotting, and staining of the blots with anti-AKAP5 antibodies. (C) HEK293 cells stably expressing GFP-tagged AKAP5 were transiently co-transfected with an expression vector harboring HA-tagged AKAP5. The cells were either untreated or incubated with a chemical inhibitor for either MEK1/2 (PD98059, 20 μM) or cyclic AMP phosphodiesterase 4 (Rolipram, 10 μM) for 45 min prior to cell lysis and analysis by SDS-PAGE. Pull-down of the HA-tagged AKAP5 was accomplished with anti-HA antibodies. The immune precipitates were subjected to SDS-PAGE and immunoblotting. The resolved, transferred protein blots were stained with anti-AKAP5 antibodies. The results displayed are representative of at least three separate experiments performed on as many separate cell cultures.

We sought to explore further the possible existence of AKAP5 dimers using somewhat less harsh conditions. HA-tagged AKAP5 was expressed transiently in HEK293 clones stably expressing GFP-tagged AKAP5 also. Pull-downs of HA-tagged AKAP5 were subjected to SDS-PAGE and immunoblotting (Figure 6A). In lysates of cells expressing two forms of exogenous AKAP5 (HA-tagged and GFP-tagged AKAP5), pull-downs of HA-tagged AKAP5 revealed the presence of the GFP-tagged AKAP (Figure 6A). This observation clearly suggests that AKAP5, like AKAP12, oligomerizes. The possibility of dimerization/oligomerization of AKAP5 was interrogated also by *in vitro *analysis of AKAP5-AKAP5 binding making use of c-Myc tagged AKAP5 (expressed and purified from yeast) in combination with His-tagged AKAP5 expressed and purified from *E. coli*. A c-Myc-tagged AKAP5/anti-Myc antibody that was coupled to protein G-agarose beads was prepared. As a control, protein G-agarose beads were similarly coupled with mouse IgG. The purified His-tagged AKAP5 was incubated with both types of derivatized protein G-agarose beads. Following incubation, the agarose beads were collected, washed, and analyzed for His-tagged AKAP5 bound to the c-Myc-tagged AKAP5 (Figure 6B). AKAP5 forms oligomers. The ability of His-tagged AKAP5 to bind immobilized AKAP5 resolves the key issue. By making use of two different approaches with conditions less harsh that those tolerated by AKAP12, we were able to establish that AKAP5 oligomerized. Pre-treating cells for 45 min with enzyme inhibitors for MEK1/2 (PD98059) and PDE4 (Rolipram) had no effect on the ability of HA-tagged AKAP5 to bind GFP-tagged AKAP5 in pull-downs of the HA-tagged version (Figure 6C), as shown by the presence of the higher *M_r _*GFP-tagged AKAP5 in the precipitate.

**Figure 7:**
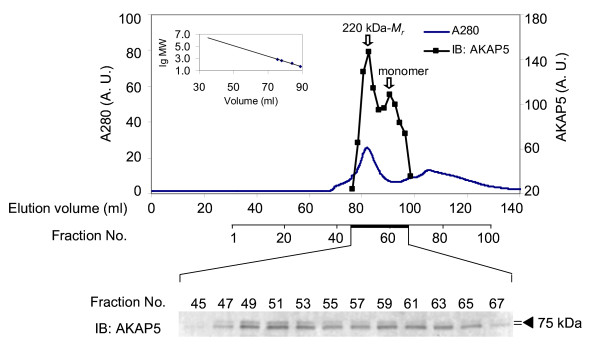
**Oligomerization of AKAP5 expressed and purified from *E. coil*: analysis of higher-order assembly by steric-exclusion chromatography**. His-tagged AKAP5 was expressed in *E. coli*. The AKAP5 was purified and then resolved by steric-exclusion chromatography performed on an AKTA FPLC fitted with HiPrep Sephacryl-S400 (16/60) column. The presence of supermolecular oligomers of AKAP5 was established by SDS-PAGE and immunoblotting of samples from the chromatography. The resolved, transferred proteins were stained with anti-AKAP5 antibodies. Marker protein mobilities were employed to establish the *M_r _*of AKAP5 by elution position (inset). The results displayed are representative of two separate experiments performed on as many separate cell cultures.

We employed again steric-exclusion chromatography on a high-resolution, wide-bore matrix operated by an AKTA FPLC to probe for AKAP5 oligomers (Figure 7). His-tagged AKAP5 was expressed and purified from *E. coli*. The purified AKAP5 was subjected to steric-exclusion chromatography on Sephacryl-S400. The chromatograms for AKAP5 and for A280 absorbance are displayed. Immunoblotting revealed a peak displaying a *M_r _*of ~220 kDa and a smaller second peak displaying a *M_r_*. of ~44kDa, the monomeric form of AKAP5 (Figure 7). Much like the supermolecular complex of AKAP12, the size of the oligomer of AKAP5 was consistent with that of an AKAP5 tetramer. When the whole-cell lysates of *E. coli *expressing His-tagged AKAP5 were subjected to the same steric-exclusion chromatography, complexes of AKAP5 were found to display higher *M_r_*, ~740 kDa (data not shown). Thus, the physical properties of purified AKAP5 include the ability to form homo-oligomeric, supermolecular complexes with *M_r _*suggestive of tetrameric organization.

## Discussion

The current work reports on new and important properties of AKAP. First, AKAP5 and AKAP12 are shown by several analytical methods to form homodimers. The homodimers of AKAP12 are so avidly bound that only in the presence of the strong chaotropic agent 8 M urea are the dimers dissociated.

Oligomers of AKAP5, less avidly bound to each other, were revealed through co-immunoprecipitation. AKAP5 is one-fourth the mass of AKAP12. Establishing the structural basis for the oligomerization of each scaffold will require additional studies. Second, AKAP5 and AKAP12 appear to form higher-order oligomers. Based upon steric-exclusion chromatography, the Mr of the AKAP5 oligomers is ~220 kDa, whereas that of the AKAP12 supermolecular complexes is ~840 kDa. The presence of higher-order oligomers for two GPCR-associating AKAPs suggests new and exciting possibilities for AKAP biology. Higher order protein-protein interactions, in which oligomers of AKAPs interact with other signaling molecules *e.g*., including GPCRs, serine/threonine protein kinases, tyrosine kinases, phosphoprotein phosphatases, cyclic AMP phosphodiesterase(s), *et al*., opens new possibilities for a fuller understanding of AKAP function. Not known is whether oligomerization of AKAPs (i.e., AKAP5 and AKAP12) precludes or includes the possibility of heterodimers/heterooligomers being formed between two different AKAPs. The presence of higher-order supermolecular complexes involving more than one AKAP would constitute a new and important dimension to AKAP biology.

The nature of the interaction that promotes the formation of oligomers of AKAPs, remains to be established. The primary sequences of AKAP5 and AKAP12 have been examined in detail with a least one startling conclusion, i.e., the structures of these molecules are predicted to be largely "natively-unordered." FOLDindex^© ^predicts intrinsic unfolding based upon the average residue hydrophobicity and net charge in the protein sequence [27,28]. Analysis using the FOLDIndex^© ^predicts human AKAP12 to be >90% natively unordered primary sequence. By the same analysis, AKAP5 displays >80% natively unordered sequence. This level of unordered structure is remarkable when one considers the number of proteins suspected to dock to AKAP12 [5,17,19]. Other members of the AKAP family, *e.g*., MAP2, also display major regions of natively unordered sequence. AKAP1, in contrast, displays much more ordered structure than AKAP12, AKAP5, or MAP2. By comparison, the Dishevelled scaffold proteins (3 isoforms in vertebrates) that operates in Wnt signaling display >85% ordered and little natively unordered structure [29]. Dishevelleds displays DIX, PDZ, and DEP domains and many docking partners also [30]. Therefore, the "natively unordered" primary sequence of AKAPs, like AKAP5 and AKAP12, must display docking sites quite unlike those found in DIX, PDZ, and DEP domains. The scaffolds are largely natively unordered, but yet must contribute to the ability of the scaffold to dock a myriad of signaling partners. Detailed studies of AKAP12 reveal the presence of the Receptor-Binding Domain for GPCRs [22], docking sites for protein kinase C [23], and for PDE4 [6] in regions that are largely considered to be "natively unordered". Reconciliation of these seemingly paradoxical observations regarding the unordered structure of AKAPs versus highly ordered structure of scaffolds like Dishevelled family members must awaited detailed structural analysis.

## Conclusion

The current study demonstrates for the first time that both AKAP5 and AKAP12 display the capacity to form supermolecular homo-oligomeric structures that likely influence the localization and function of these molecular scaffolds. Thus AKAP oligomerization adds a new dimension on how members of this class of scaffold molecules operate in cell signaling.

## Materials and methods

### Reagents

Mouse anti-AKAP5, goat anti-mouse IgG-HRP, and goat anti-rat IgG-HRP were purchased from Santa Cruz Biotechnology (Santa Cruz, CA). Mouse anti-AKAP12 monoclonal antibody was from Abcam (Cambridge, MA). Rat anti-HA antibody and HA-agarose beads were products of Roche (Indianapolis, IN). Rabbit anti-β-catenin antibody was from Sigma-Aldrich (St. Louis, MO). Rolipram was from Cayman Chemical Company (Ann Arbor, MI). PD98059 was a product of Millipore (Billerica, MA).

### Cell lines

The human embryonic kidney cell line HEK293 [6,16,18,31] was obtained from ATCC (Bethesda, MD) and cultured in Dulbecco's modified Eagle's medium (DMEM) containing 10% fetal bovine serum in a humidified atmosphere containing 5% CO_2 _at 37°C. Confluent cells were treated with 10 μM isoproterenol (Iso) in DMEM, for indicated times.

### Plasmids and transfection

pcDNA3.1 vector harboring HA-tagged AKAP12, HA-AKAP12M3 (S627A, S696-698A, S772A), HA-AKAP12 (1-938), and HA-AKAP12 (840-1782) were constructed as described previously (*23*). pCMV-HA vector harboring AKAP5 was generated by PCR using specific primer pairs 5'-GGAATTCGGATGGAAACCACAATTTCAGAAATTC-3' and 5'-CCGCTCGAGTCACTGTAGAAGATTGTTTATTTTATTATC-3'. The PCR products were subcloned into pCMV-HA between EcoRI and Xho I sites. To generate His-tagged AKAP12(840-1782), AKAP12(1-840) and AKAP5, the following primer pairs were employed 5'-GGAATTCGATGGGCGCCGGGAGCTCCAC-3' and 5'-CCGCTCGAGGTCATCTTCGTTGGCCCCTG-3', 5'-CGCGGATCCGGACTCTGATGTCCCGGCCGTGGTC-3' and 5'-CCGCTCGAGAGATTCTGTAAGTTCTGACTTTGC-3'; 5'-CGCGGATCCGATGGAAACCACAATTTCAGAAATTC-3' and 5'-CCGCTCGAGCTGTAGAAGATTGTTTATTTTATTATCATC-3' for PCR. The PCR products were inserted into pET28b(+) (Novagen, Merck KGaA, Darmstadt, Germany) vector. pGBKT7 vector (expresses proteins fused to amino acids 1-147 of the GAL4 DNA binding domain (BD), c-Myc tag) harboring AKAP5 was generated by PCR using primer pair 5'-GGAATTCATGGAAACCACAATTTCAGAAATTCATG-3' and 5'-TGACGCGTCGACTCACTGTAGAAGATTGTTTATTTTATTATC-3'. The PCR product was subcloned into pGBK-T7 vector between EcoR I and Sal I sites. HEK293 cells were transfected with plasmid DNA using ExpressFect (Denville Scientific, Metuchen, NJ), according to the manufacturer's instructions.

### Purification of His-tagged AKAP5 and AKAP12

His-tagged AKAP5, AKAP12 (840-1782) and AKAP12 (1-840) expressed in *E. coli *were purified by using Ni-NTA matrix (Qiagen, Valencia, CA), according to the manufacturer's protocol. His-tagged AKAP12 was purified by using Superose 12 column (12/29.5, GE Healthcare) pre-equilibrated with 20 mM Tris, 0.2 M NaCl and 10% glycerol. Fractions containing AKAP12 were collected.

### In vitro analysis of AKAP5 dimerization

c-Myc-tagged AKAP5 fused to amino acids 1-147 of the GAL4 DNA-binding domain (BD) was expressed in yeast and pulled down from yeast lysates with anti-c-Myc antibody. The c-Myc-tagged AKAP5/antibody complex was absorbed to protein G-agarose beads. Protein G-agarose beads similarly treated with mouse IgG served as a control for this step. His-tagged AKAP5 was expressed and purified from *E. coli*, as above. The purified His-tagged AKAP5 was incubated at 25°C for 1 h in PBS containing 1% Triton X-100 and 1 mg/ml BSA in combination with protein G-agarose beads to which either c-Myc-tagged AKAP5 or control IgG were bound. At the end of the incubation, the protein G-agarose beads were collected and washed in PBS containing 1% Triton X-100 three times; the beads then were collected and heated with 30 μl Laemmli buffer at 95°C for 5 min. The supernatant was subjected to SDS-PAGE. AKAP5 was detected by immunoblotting of the resolved proteins using anti-AKAP5 antibody.

### Steric-exclusion chromatography of AKAP supermolecular complexes

Purified His-tagged AKAP5 or AKAP12 was applied to the Sephacryl-S400 gel filtration column (HiPrep Sephacryl S-400 High resolution 16/60, GE healthcare) making use of a fast-performance liquid chromatography system AKTA (GE Healthcare), pre-equilibrated with PBS supplemented with 0.01% NaN_3_. Sample (1.0 ml) fractions were collected. Each fraction was analyzed by SDS-PAGE and immunoblotting. Protein was detected in the flow-cell by measuring absorbance at A280.

### Immunoprecipitation and immunoblotting

Cells were harvested in a lysis buffer containing 20 mM Tris-HCl, pH 7.4, 1% Nonidet P-40, 2 mM sodium orthovanadate, 150 mM NaCl, 5 mM EDTA, 50 mM NaF, 40 mM sodium pyrophosphate, 50 mM KH_2_PO_4_, 10 mM sodium molybdate, and a cocktail of protease inhibitors (Complete Protease Inhibitor Cocktail tablet, Roche, Nutley, NJ). After centrifugation at 10,000 × *g *for 15 min, the protein concentration of the supernatant was determined using the Bradford reagent. One mg of protein was incubated with specific primary antibody for 4 h at 4°C, then 20 μl of protein A/G agarose was added and the mixture was incubated for 2 h on a rolling mixer. Immune complexes were collected and washed three times with PBS containing 1% Triton X-100, and thereafter boiled for 5 min at 95°C in 30 μl of Laemmli buffer. The supernatant was subjected to SDS-PAGE and the resolved proteins were transferred to a PVDF membrane. The blots were probed with specific antibodies against target proteins. The immune complexes on the blots were made visible by staining with a horseradish peroxidase-conjugated secondary antibody in combination with the chemiluminescence reagent, followed by a brief autoradiography using autoradiography film (WorldWide Medical products Inc, Hamilton, NJ).

## Abbreviations

AKAP: A-kinase Anchoring Protein; DMEM: Dulbecco's modified Eagle's medium; A431: human epidermoid carcinoma cells; HEK293: human embryonic kidney 293 cells; PKA: Protein kinase A; PDE: Phosphodiesterase; SDS-PAGE: sodium dodecyl sulfate-polyacrylamide gel electrophoresis; SEC: steric-exclusion chromatography

## Competing interests

The authors declare that they have no competing interests.

## Authors' contributions

SG collected the data and wrote the draft manuscript; HYW and CCM edited the draft manuscript and figures of the final version of this unpublished work. Each author read and approved the final manuscript.
